# Seasonality and uncertainty in global COVID-19 growth rates

**DOI:** 10.1073/pnas.2008590117

**Published:** 2020-10-13

**Authors:** Cory Merow, Mark C. Urban

**Affiliations:** ^a^Eversource Energy Center, University of Connecticut, Storrs, CT 06268;; ^b^Center of Biological Risk, University of Connecticut, Storrs, CT 06268;; ^c^Department of Ecology & Evolutionary Biology, University of Connecticut, Storrs, CT 06268

**Keywords:** SARS-CoV-2, climate, pandemic, UV light, humidity

## Abstract

The virus causing COVID-19 has spread rapidly worldwide and threatens millions of lives. It remains unknown, as of April 2020, whether summer weather will reduce its spread, thereby alleviating strains on hospitals and providing time for vaccine development. Early insights from laboratory studies and research on related viruses predicted that COVID-19 would decline with higher temperatures, humidity, and ultraviolet (UV) light. Using current, fine-scaled weather data and global reports of infections, we develop a model that explains 36% of the variation in maximum COVID-19 growth rates based on weather and demography (17%) and country-specific effects (19%). UV light is most strongly associated with lower COVID-19 growth. Projections suggest that, without intervention, COVID-19 will decrease temporarily during summer, rebound by autumn, and peak next winter. Validation based on data from May and June 2020 confirms the generality of the climate signal detected. However, uncertainty remains high, and the probability of weekly doubling rates remains >20% throughout summer in the absence of social interventions. Consequently, aggressive interventions will likely be needed despite seasonal trends.

## Significance

The virus causing COVID-19 has spread rapidly worldwide. It remains unknown whether summer weather will reduce its spread and justify relaxing political interventions and restarting economic activities. We develop statistical models that predict the maximum potential of COVID-19 worldwide and throughout the year. We find that UV light, in particular, is associated with decreased disease growth rate relative to other analyzed factors. Based on these associations with weather, we predict that COVID-19 will decrease temporarily during summer, rebound by autumn, and peak next winter. However, uncertainty remains high, and many factors besides climate, such as social interventions, will influence transmission. Thus, the world must remain vigilant, and continued interventions will likely be needed until a vaccine becomes available.

COVID-19 is causing widespread morbidity and mortality globally (1, 2). The severe acute respiratory syndrome coronavirus 2 (SARS-CoV-2) responsible for this disease infected more than 17 million people by August 2020 (3). Much of the world has implemented nonpharmaceutical interventions, including preventing large gatherings, voluntary or enforced social distancing, and contact tracing and quarantining, in order to prevent infections from overwhelming health care systems and exacerbating mortality rates (2, 4). However, these interventions risk substantial economic damage, and thus decision makers are currently developing or implementing plans for lifting these restrictions. Consequently, improved forecasts of COVID-19 risks are needed to inform decisions that weigh the risks to both human health and economy (2).

One of the greatest uncertainties for projecting future COVID-19 risk is how weather will affect its future transmission dynamics. SARS-CoV-2 might be particularly sensitive to weather, because preliminary laboratory trials suggest that it survives longer outside the human body than other viruses (5). Rising temperatures and humidity in the Northern Hemisphere summer could reduce SARS-CoV-2 transmission rates (6–8), providing a temporary reprieve. Simultaneously, the Southern Hemisphere has entered winter, and we do not know whether winter weather will increase COVID-19 risks, especially in countries with reduced health care capacity. Early analyses of COVID-19 cases predicted that high temperatures would reduce summer transmission (9–11). These predictions have been widely reported and are informing decisions about relaxing interventions. However, these analyses relied on the early stages of viral spread before the epidemic had reached warmer regions and thus potentially conflated weather with initial emergence and global transport.

We estimate how weather affects COVID-19 growth rate using data from the first four months of pandemic spread (up to April 13, 2020) when social interventions were rare and apply methods that improve model predictive accuracy, incorporate uncertainty, and reduce biases. We developed several predictions about how weather, either directly or indirectly via modified human behaviors (e.g., aggregating indoors) or effects on immune function, affects COVID-19 growth rate based on a literature review of weather impacts on SARS-CoV-2 (9, 10, 12), related coronaviruses (8, 13–15), and viruses involved in other epidemics such as influenza (16–19). Based on this research, we predicted that COVID-19 growth would peak at low or intermediate temperatures. Alternatively, other coronaviruses demonstrate weak temperature dependence, instead depending on social or travel dynamics (7). High humidity also might decrease viral persistence, limit transmission of expelled viral particles, or decrease host resistance (13, 20–23). Ultraviolet (UV) light effectively inactivates many viruses (19), especially larger coronaviruses (24) like SARS-CoV-1 (25). Sunny days might decrease outdoor transmission or promote immune resistance via vitamin D production (26). We also evaluate demographic variables, hypothesizing greater transmission in denser (more interactions among people) and older (>60 y) populations that are more likely to have severe symptoms and be tested as compared to less symptomatic populations of younger people.

We modeled maximum growth rates of COVID-19 cases to restrict analyses to the early growth phase before social interventions reduced transmission, but after community transmission began, and when most people were still susceptible to this novel virus. We estimated the average maximum growth rate (λ) as the exponential increase in cases (ln N_*t*_ – ln N_0_)/*t*, where N_*t*_ = cases at time, *t*, and N_0_ = initial cases) based on a repeated measures design for the three worst 1-wk intervals in each political unit [country or state/province, depending on available data (3)], where *t* = 7 d. We chose a 7-d period because of evidence that case reporting varies significantly by day of week, with a weekly cycle in global data described by a peak on Friday and a low on Monday (*SI Appendix*, Fig. S5). Moreover, an analysis of temporal autocorrelation in detrended data revealed significant peaks at 7 and 14 d, again corroborating a weekly pattern to reporting that is overcome by using a 7-d period (*SI Appendix*, Fig. S5). However, we also evaluated results at shorter periods and found that results were also robust to using 1- or 3-d intervals (*SI Appendix*, Fig. S6). Testing and reporting of COVID-19 varies considerably across political units, which makes modeling differences in raw count data unreliable. In contrast, estimated growth rates should remain robust to the biases introduced by variable reporting rates, assuming detection probabilities remain relatively similar during the short, 1-wk estimation period in a given political unit. Although data on testing rates to evaluate this assumption are unavailable for many political units, we believe that this approach is the least biased approach given data limitations. We restricted analyses to political units with >40 cases, to eliminate periods before local community transmission. These decisions resulted in data from 128 countries and 98 states or provinces.

We applied a hierarchical Bayesian model with uninformative priors to estimate parameters. The Bayesian approach provides a transparent means to account for and explore uncertainty through the posterior distribution of estimates, and thus has become the preferred methodology of many forecasting studies. We obtained daily infection data from ref. 3 and obtained 3-h weather data from the European Centre for Medium-Range Weather Forecasts Re-Analysis model (ERA5) reanalysis for the 14-d preceding case counts and averaged these values to reflect the possibility that infection could have occurred during the previous 14 d, consistent with the 1- to 14-d infective period widely reported (27). Given the uncertainty in the joint distributions of symptom onset, testing, and reporting, as well as not knowing the degree to which variables influenced COVID-19 case growth via transmission versus the expression of symptoms (e.g., vitamin D immune function), we chose to average across the potential period of infectivity, thereby assuming weather each day in the preceding 14 d was equally important. However, results were robust to a range of other assumptions when calculating lagged weather variables, including weighted means centered on 6, 9, and 12 d as well as different variances. We demonstrated that the insensitivity of results to these assumptions originated from the high similarity of weather variables over 14 d in each political unit relative to their dissimilarity among political units (see *SI Appendix*, Fig. S7 for more information). We used fine-scaled weather data rather than long-term climatic monthly means to model observed weather outbreak dynamics. Weather data were weighted by population size in each 0.25° grid cell within each political unit to capture the weather most closely associated with outbreaks in population centers. We used leave-one-out cross-validation, which ranks models on predictive accuracy on excluded data, to choose the highest performing models. We included a random country effect to account for differences in national control response times, health care capacity, testing rates, and other characteristics intrinsic to country of origin.

**Fig. 1.**
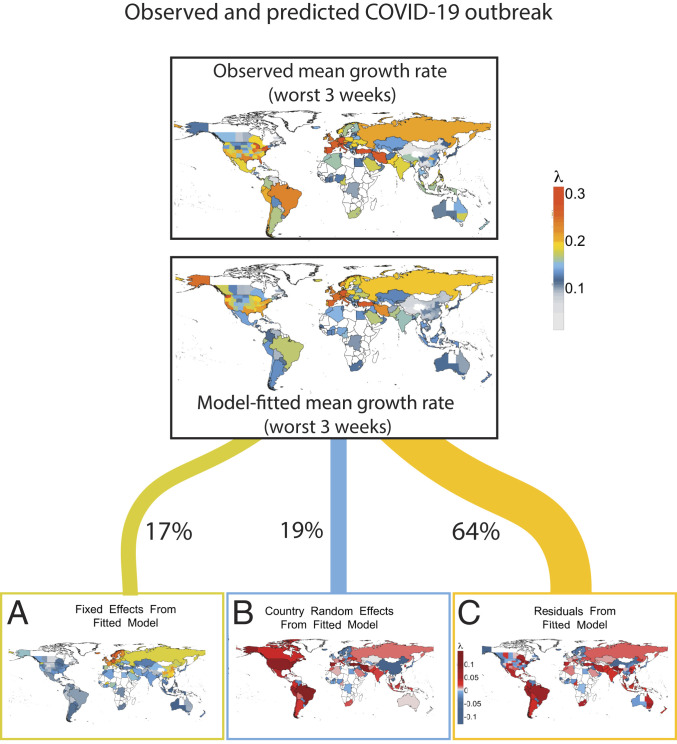
Observed and predicted maximum growth rates for COVID-19 along with graphical partitioning of (*A*) weather and demography, (*B*) country effects, and (*C*) residual variation. Country effects are estimated relative to global mean; 17% of variation is explained by seasonality, while 19% of variation arises from country-specific factors, which may include quarantine policies, health care, or reporting practices.

**Fig. 2.**
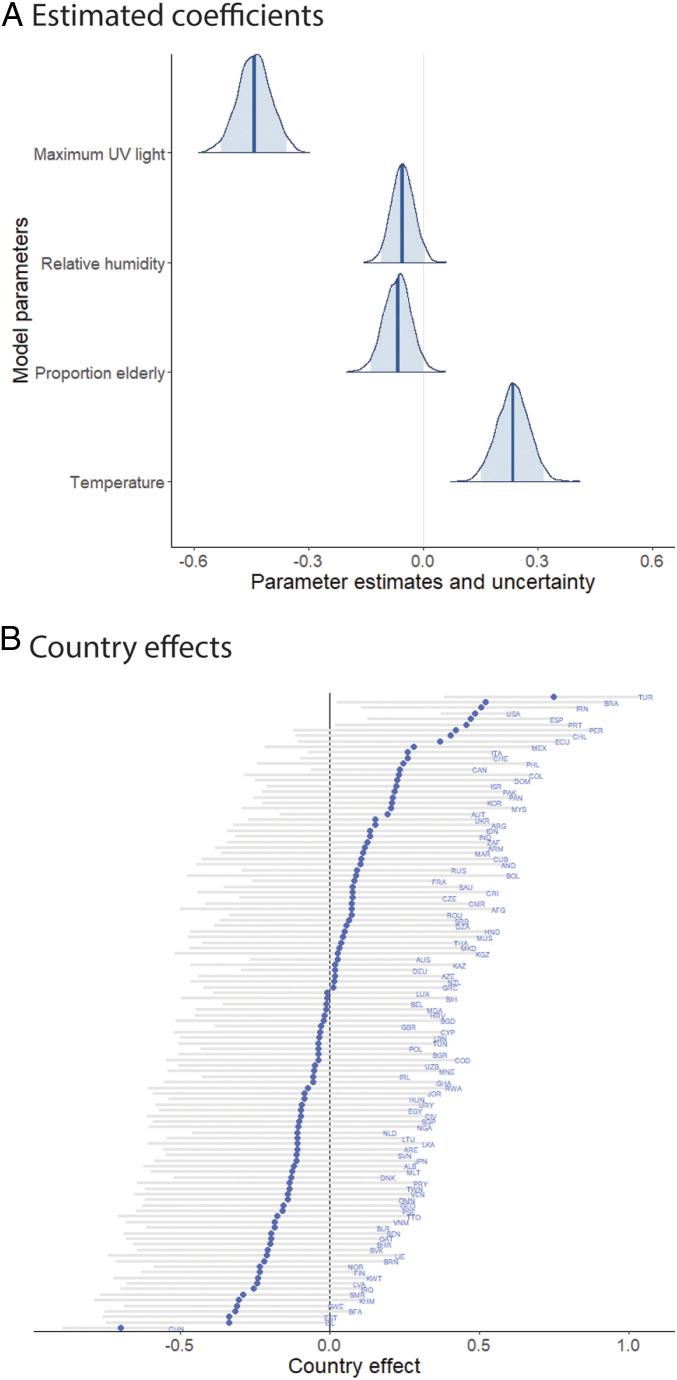
Median standardized estimates for (*A*) weather and demography and (*B*) country effects for best predictive model with 95% Ci (light blue) and medians indicated by dark blue vertical lines or diamonds, respectively. Country codes follow GADM (Global Administration) conventions.

The best model predicted 36% of the variation in maximum COVID-19 growth rate (Fig. 1) and 17% of the variation including weather and demographic variables, but excluding country effects. This model included maximum daily UV light, mean daily temperature, proportion of elderly, and mean daily relative humidity (Fig. 2*A*). Competing models reflected the same qualitative results and similar parameter estimates (*SI Appendix*, Table S1).

UV light had the strongest and most significant effect of tested meteorological variables on COVID-19 growth (*β*_*UV*_ = −0.44, 95% credible interval [Ci]: −0.53, −0.36; covariates were standardized to allow for comparisons among coefficients). Many other human viruses also peak during periods of low UV light in winter, including influenza (19). This negative effect of UV light on SARS-CoV-2 might be even stronger given evidence that the large genome size of coronaviruses makes them particularly susceptible to sunlight-derived UV irradiation (25). Alternatively, UV light might decrease COVID-19 risk indirectly by facilitating human immunity function by enhancing vitamin D production. Several reports now suggest a link between vitamin D deficiency and increased risk of COVID-19 (26, 28, 29).

Contrary to predictions, temperature positively affected COVID-19 growth rate (*β*_*temp*_ = 0.23, 95% Ci: 0.15, 0.32), when combined with UV light (see *SI Appendix* for discussion of alternative candidate models and effects of variable correlations). Like most viruses, SARS-CoV-2 likely performs best in a moderate range of temperatures, such that, when combined with correlated factors such as UV light and humidity, our model incorporates the positive aspects of this unimodal relationship. As expected, relative humidity decreased growth rates (*β_humidity_* = −0.05, 95% Ci: −0.11, 0.00). Absolute humidity was strongly correlated with temperature (*r* = 0.88), and adding it made little difference in model performance. Humidity is frequently an important factor in reducing the transmission of viral particles through the air, as demonstrated for the influenza virus (22, 23).

Contrary to predictions, the proportion of elderly in a population was associated with decreased COVID-19 growth rate (*β*_*prop_over60*_ = −0.07, 95% Ci: −0.14, −0.00), perhaps due to outbreaks facilitated by early transmission in northern temperate countries with older populations or risk-averse behaviors in older populations. The model was characterized by equally strong random effects associated with country of origin (Fig. 2*B*). For instance, Turkey, Brazil, Iran, and the United States had the highest growth rates independent of modeled factors, whereas China, Iceland, Burkina Faso, and Sweden had the lowest. The strong negative effect associated with China likely indicates early interventions and is accounted for in our model.

**Fig. 3.**
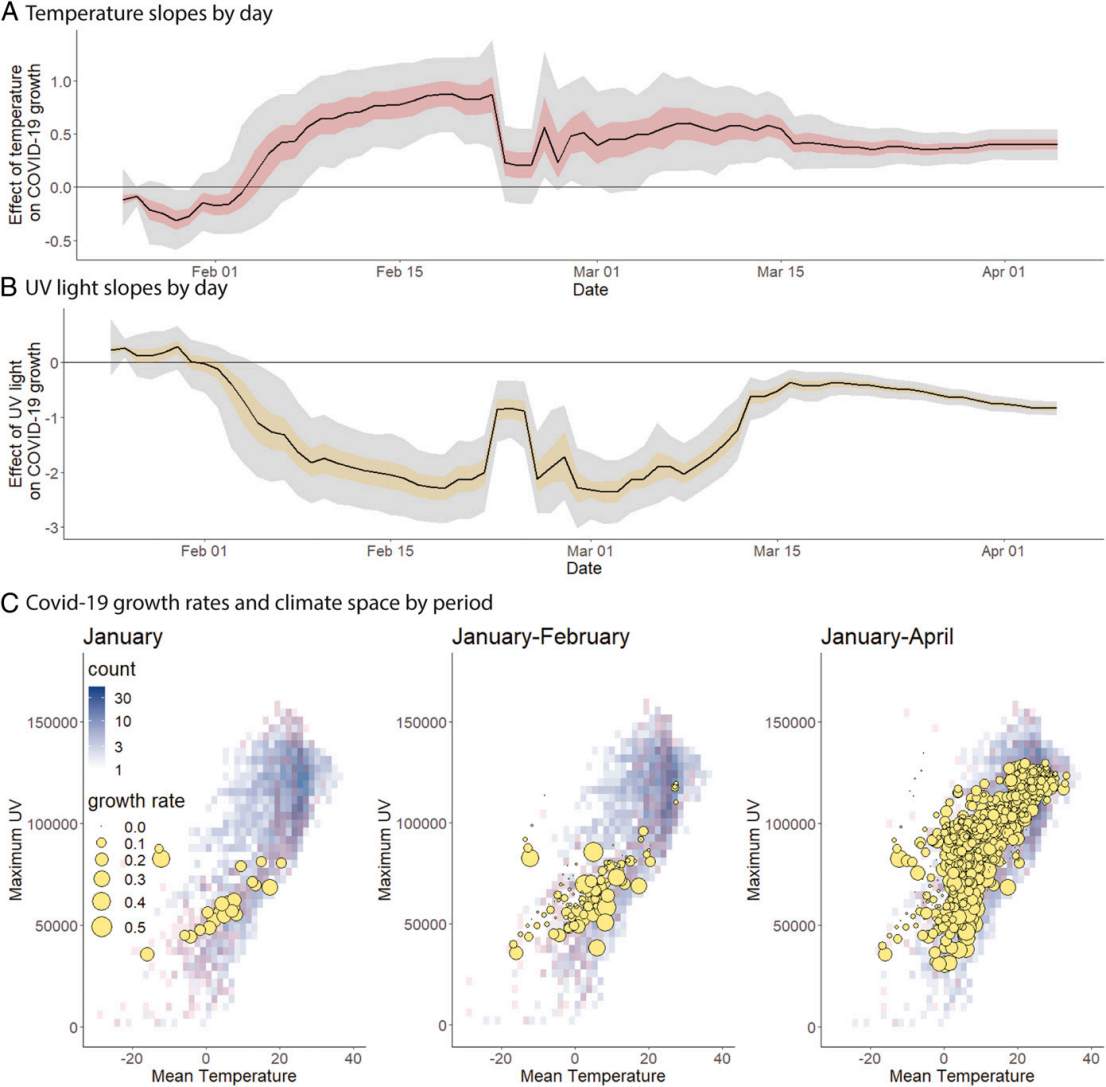
Effect of temperature and UV on COVID-19 growth rate as the pandemic spreads to new climates. (*A* and *B*) Model coefficients and uncertainty through time demonstrate dynamic shifts and stabilization of parameter estimates (50% and 95% Ci indicated by colored and gray fills, respectively), illustrating why earlier studies likely detected a negative temperature dependence. China dominated data until February 24, after which COVID-19 spread to new regions and novel climate space, leading to the observed shift in model coefficients. (*C*) Early COVID-19 outbreaks (growth rate proportional to blue symbol area) occurred in a subset of potential temperatures (degrees Celsius) and UV light levels (joules per square meter) possible per year (background gray-blue gradient) and per time period (red overlay) based on 2014–2019 climate averages.

We explored why earlier studies predicted a negative association between temperature and COVID-19. Alone, temperature had a weak, negative effect on COVID-19 growth rate, but this effect became positive after adding UV (*SI Appendix*, Table S1). When combined with other parameters, temperature negatively affected COVID-19 early in the pandemic (Fig. 3, *Top*). Significant positive temperature dependence emerges by late February following transmission to warmer, high-UV regions of climate space, like Africa (30) (see Fig. 3, *Bottom*, demonstrating filling of climate space). Note, however, that our analysis does not specifically attempt to reproduce previous studies, so differences are expected depending on the details of decisions in other studies. This finding urges caution in drawing conclusions about the climatic niches of new pathogens from initial emergence sites and transportation hubs before they reach an equilibrium distribution with climate. Although future data could also alter our predictions, especially as COVID-19 becomes endemic (31), we found evidence that model predictions might have stabilized by the end of the analysis. This evidence includes less variable model predictions over time (Fig. 3*A*) and the filling of available global climate space such that COVID-19 is now found in most available climates of the world, and therefore relationships are likely to reflect the multitude of possible global weather patterns rather than the subset of weather where it originated (Fig. 3*B*).

**Fig. 4.**
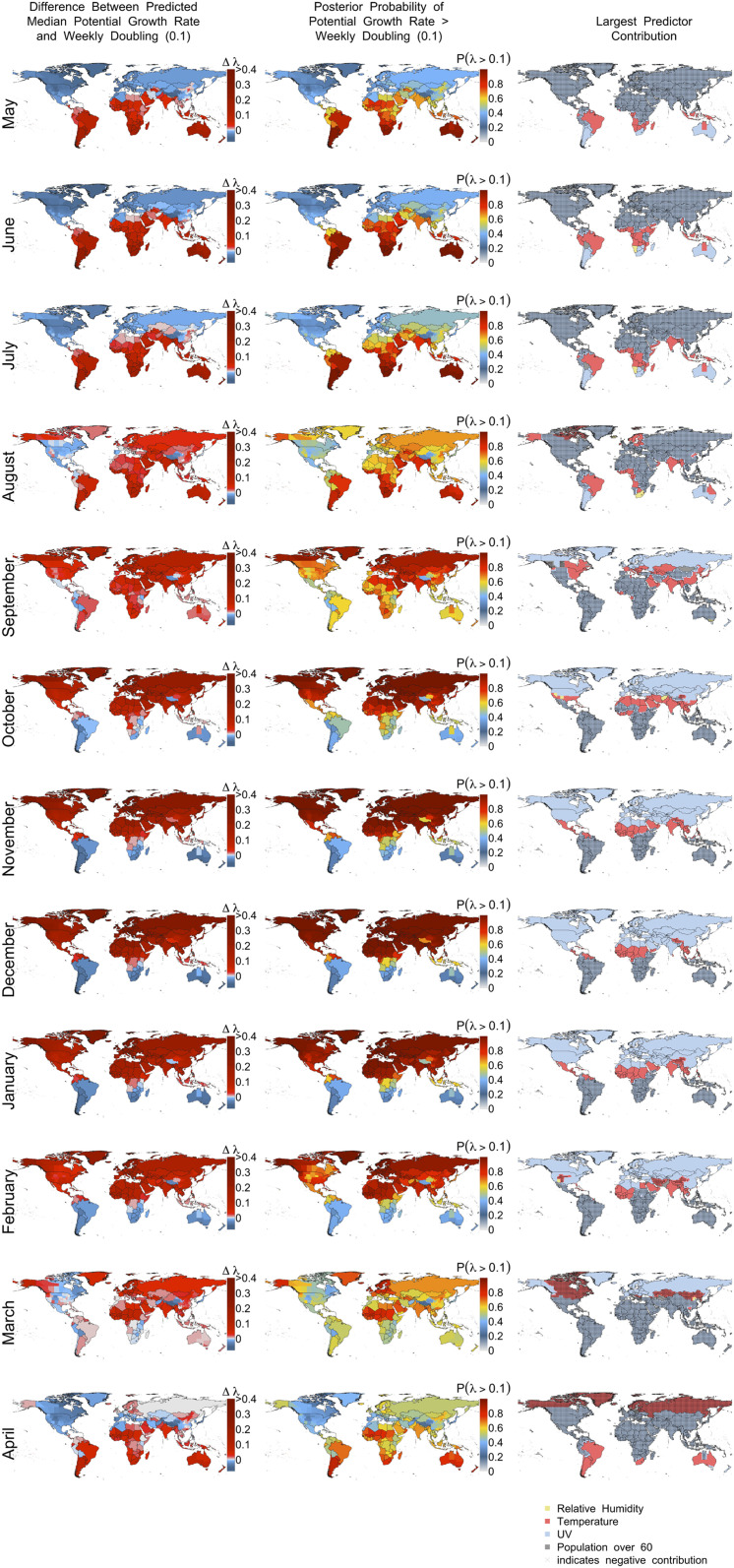
Predicted potential growth rates of COVID-19 by month using best model. (*Left*) Potential growth rate relative to weekly doubling time (*λ* = 0.1). Red indicates faster than a weekly doubling rate, and blue indicates slower rates. (*Middle*) Posterior probability of growth rates exceeding a weekly doubling rate. (*Right*) Indicates which predictor contributes most (based on predictor × coefficient) to COVID-19 growth rate (*λ*) in each 0.25° cell, with stippling indicating negative contributions.

In April 2020, we predicted potential COVID-19 growth rates for the next 12 mo relative to a weekly doubling rate (*λ* = 0.1; Fig. 4). Based mostly on contributions from UV light and temperature, our model predicts that COVID-19 risk will decline—although it will not be eliminated—across the Northern Hemisphere this summer, remain active in the tropics, and increase in the Southern Hemisphere as days shorten and UV light declines (Fig. 4, *Left* and *Right*). However, given high uncertainty, a nonnegligible risk exists throughout the world for potential outbreaks in summer similar to that observed at the outset of the pandemic (Fig. 4, *Middle*, dark blue = 30% probability of *λ* > 0.1). By September, declining daylength steadily increases COVID-19 outbreak risk in the Northern Hemisphere until a peak in northern winter (December−January), while risks decline during the Southern Hemisphere’s summer. This predicted peak of COVID-19 in winter corresponds to what we know about coronaviruses more generally. For instance, a study of multiple human coronaviruses in southern China found that they also peaked in winter (15).

**Fig. 5.**
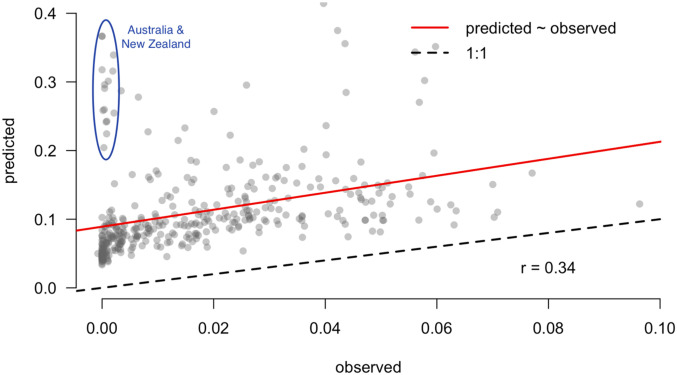
Predictions were validated with data from May and June 2020 that became available after the model was parameterized (in April 2020). Predictions generally follow a linear trend (red line) parallel to the 1:1 line (black line), but with a positive offset (higher intercept) that shifts the trend line up. We hypothesize that this offset reflects the effect of recent, widespread interventions that were not as prevalent during model development. Despite this offset, the similar slope of the relationship suggests that the model still retains predictive power, even during robust social interventions. Outliers are shown in the blue circles, and occur primarily in Australia and New Zealand, where interventions have been largely successful, and, to a lesser extent, in central South America.

Predictions should not just be developed, but should ultimately be validated with out-of-sample data in order to test them (32, 33). During the process of revising this manuscript, it became possible to validate predictions made in early April 2020 with data that became available for May and June 2020. The new data were aggregated into weekly intervals for each polity to calculate weekly growth rates that were comparable to our original predictions. We then took the average value over weeks within a month and compared that to the values of predictions shown in Fig. 4 for each polity. Because the model was fit during a period of limited intervention in most regions and the validation data include a time period when interventions are high in most regions, we hypothesized downward-biased estimates. However, we expected a correlation between predictions and observations (i.e., consistency) due to weather, and found a correlation of 0.33. The considerable scatter that remains (Fig. 5) is consistent with our expectation that human behaviors have larger impacts on spread than climate. However, there remains a clear signal that the climate relationships we detected are reflected in validation data.

Although this model represents our best current prediction, a range of outcomes still remain possible within the scope of our uncertainty estimates (Fig. 4, *Middle Column*). Furthermore, these predictions of potential growth need not be realized if appropriate interventions are enacted or a vaccine is developed. Whereas intervention will substantially influence absolute growth, our predictions can still inform our understanding of the underlying annual variation in risk. For instance, the flu still cycles seasonally and from hemisphere to hemisphere despite availability of seasonal flu vaccines (17). Although not conclusive, the decline in COVID-19 growth rate in the Northern Hemisphere combined with its rapid growth in South America and South Africa in June is consistent with predictions made in April and despite the maintenance of strong social interventions up to this point in time.

Our predictions were robust to the manifold decisions made regarding data and model structure. We explored the consequences of using different parameter comparisons, the effects of 7-d periods for aggregating weather data, different cut-offs for minimum number of cases, varying number of weeks analyzed per political unit, analyzing first or worst weeks following the infection threshold, and using weather maxima and minima instead of means. In all cases, we found no qualitative changes to results, except that maximum daily UV light during a 14-d interval substantially outperformed the mean (*SI Appendix*), which was subsequently included in the top model. We also explored the effect of excluding data from China, where interventions occurred first, and found qualitatively similar results. Lastly, we evaluated the effect of spatial and temporal autocorrelation on model estimates and found little support for its impacts on model results (*SI Appendix*, Figs. S3 and S4).

Understanding the true contributions of weather to human pathogens requires combining insights from observational analyses like this one and manipulative experiments that isolate factors under controlled conditions (5, 12). Other causal factors correlated with weather variables could have also contributed to our findings, including weather-associated human behaviors (e.g., seasonal aggregations for education or religion). Despite initial suggestions that seasonality would substantially control COVID-19, we found that weather explains 17% of the variation in COVID-19 growth rates. Undescribed factors across political units were as important as weather (19% of variation), and much of the variation (64%) remains unexplained. Future studies should build from these meteorological insights and create more mechanistic epidemiological models that include laboratory-based estimates of weather impacts, human demography, movement, sociocultural behaviors, health care capacity, and political interventions (e.g., refs. 2, 4, and 31).

Other epidemiological factors are likely to explain more variation in epidemic growth rates beyond weather and demography. For example, each of the 10 influenza outbreaks in the past 250 y have peaked 6 mo after the start, regardless of the season in which they began. Although the dynamics of influenza and SARS-CoV-2 may differ, this recurrent pattern likely occurs due to the high susceptibility of populations to new viruses, as has been suggested for SARS-CoV-2 (31). Yet, 17% of variation explained by weather is still exceptional compared to meteorological models of viral outbreaks such as seasonal influenza (17, 18, 34), in which weather only explained 3% or less of variation. In contrast, a model of the related SARS-CoV-1 virus explained 85% of the variation in transmission based on weather. Laboratory research has suggested that coronaviruses might persist longer in the environment (5) and are more susceptible to UV inactivation (24). Together, these pieces of information suggest that SARS-CoV-2 might be more sensitive to weather and thus might display more climate-driven seasonality than other viral epidemics. However, we reinforce that effective pharmaceutical and nonpharmaceutical interventions are far more important in altering outbreak dynamics, with seasonality operating as a contributing underlying factor.

We demonstrated and validated that COVID-19 growth rate increases with reduced UV light, higher temperatures, and lower relative humidity. We predict that COVID-19 will oscillate between the Northern and Southern Hemispheres, based largely on seasonal variation in UV radiation and temperature without continuing interventions like social distancing. Despite a possible, but uncertain, temporary summer reprieve in the north, COVID-19 will likely return by autumn and threaten further outbreaks. The north should take this time to build resilience against future outbreaks, while assisting countries in the tropics and Southern Hemisphere. Uncertainty remains high, however, so we urge caution when making decisions such as removing societal interventions before more permanent pharmaceutical solutions can be implemented.

## Methods

### Overview.

We examined the weekly rate of increase in the number of COVID-19 infections as a function of weather, while controlling for human population structure, in order to determine the effects of the abiotic environment on the growth rate of infections. Our selection of weather variables and the time frame within which we measured variation was based on the limited, but rapidly expanding, experimental and observational research on the survival and transmission of SARS-CoV-2 virus and human resistance to the resultant COVID-19 disease (9, 12, 26, 31, 35). We performed model selection to optimize model prediction of cross-validated data and performed comprehensive sensitivity analysis with respect to both data preparation and modeling decisions and found no qualitative differences between the findings represented in our best model and other models using different, but reasonable, decisions.

### Infection Data.

Daily infection data were obtained from the Johns Hopkins Center for Systems Science and Engineering (3), which documents country-level aggregations of infected individuals, except in Australia, Canada, China, and the United States, where state-level data are available. From these daily data, we calculated weekly growth rate assuming an exponential model for the growth of the number of infected individuals, which fit well to COVID-19 dynamics during the early stages of spread. The starting point for 1-wk intervals was polity specific (either country or state level depending on the resolution of available data), and calculated beginning on the first day (denoted *t*_0_) that the number of infected individuals exceeded 40 (and 20 and 60; see sensitivity analysis below). This minimum was necessary to eliminate the early dynamics of COVID-19 in locations due primarily to transport from other regions rather than local, community transmission. This moving window approach allowed us to capture local differences in onset date of transmission without imposing any artificial cutoffs (e.g., based on calendar week). By summarizing the data in this way, we had 541 observations distributed over 203 political units.

To capture periods when the spread rate was most severe, we chose to focus on the worst 3 wk (also 2, 4 wk; see sensitivity analysis) in each political unit based on the magnitude of lambda. We were primarily concerned about high rates of spread and their possible meteorological and demographic drivers, so this decision controls for differences among polities in the onset of severe spread and differences in the timing of control measures that may reduce growth. Hence, a focus on maximum growth rates is the best, unbiased estimate of COVID-19 growth in the absence of control measures and most likely to be influenced by weather. In sensitivity analyses, we also considered using the first 2, 3, or 4 wk following *t*_0_, and found similar, but more variable, results, owing to the likely variation among countries in the early rates of spread (e.g., in Thailand, growth was initially low before increasing rapidly).

### Weather Data.

Weather data were aggregated from 3-hourly data downloaded from the ERA5 model (36) and averaged at 14-d intervals preceding the time period in which they were calculated for each polity. A 14-d interval captures the known infective period of SARS-CoV-2, where infections are known to occur from a period of 1 d to 14 d (27). Hence, we use the actual observed weather during the period of viral transmission. This decision contrasts with previous studies that used average monthly climate calculated over the interval 1970–2000 provided by Worldclim (37). Notably, the biweekly averages we calculated are, on average, expected to reflect higher temperatures due to climate change in the last 50 y compared to historic long-term averages. Further, our biweekly estimates better reflect the actual conditions when infections occurred, and thus are expected to better predict transmission if, indeed, they influence it.

Based on existing insights about SARS-CoV-2 and the onset of COVID-19, we considered the following weather variables: temperature 2 m above land surface, relative humidity, absolute humidity, and total incoming UV radiation at the land surface. To align the weather data with infection data for a given political unit, we determined the first day (*t*_0_) when more than 40 individuals were reported (also 20 and 60 infections; see below). We calculated the mean values of the weather variables over the 14-d window preceding t_0_. For example, *t*_0_ for Connecticut was March 16 (when 41 records had accumulated), so the weather variables were averaged over the 14-d window preceding March 10. This reflects the assumption that detected infections between March 10 and March 16 primarily occurred between February 24 and March 9. Although imperfect, the temporal autocorrelation of weather suggests that this is reasonable (e.g., even if an infection occurred on March 2, weather would be typically well correlated from March 3 to March 9).

Finally, note that we also explored the use of minimum and maximum values of weather variables to account for the possibility that transmission was more likely driven by extreme weather rather than average weather. We also considered using weekly rather than biweekly intervals, to reflect the possibility of shorter incubation periods. Outcomes were robust to these decisions (*SI Appendix*, Table S1).

Previous studies have noted that the coarse spatial grain of infection data (country or state level) makes it difficult to interpret weather variables in the context of such large spatial units (38). To address this, we calculated weather averages over the quarter-degree grid cells in a polity, weighted by the population size in each cell. This resulted in weather covariates that better reflect where most humans are and hence where infections occurred. Also, early maximum transmission rates were usually located in large cities, and thus our model weights weather variation in line with this potential bias.

### Population Data.

We obtained human population data from Worldpop.org, focusing on total human population (density) and proportion of the population over age 60 y. Population density was hypothesized to control for the number of interactions individuals in a location were likely to experience, whereas the proportion of people over age 60 y in a polity was hypothesized to control for reporting rate, given that older people are more adversely affected by the disease and thus more likely to be tested. Data were obtained at 1-km resolution and summed to the quarter-degree grid imposed by the weather data. Polity information was obtained based on global standards (GADM.com). Each quarter-degree grid cell was assigned to a polity, and cells were averaged over the polity.

### Models.

We focus on the growth rate of COVID-19 cases, rather than estimating a climate niche for the virus based on its presence or absence or total number of cases, as explored in preliminary studies (11), to avoid issues with disequilibrium in the virus’ distribution. We focused on estimating the rate of increase of infected individuals, rather than directly modeling the number of infected individuals, in order to minimize the influence of different reporting biases in different polities. We calculated *λ* as *λ* = (ln(*N*(*t*)) − ln(*N*(*t*_*0*_))/*t*, where *t* was taken to be 7 d and *t*_*0*_ was defined as the start date for counting infections. This formulation is independent of reporting bias, under the assumption that the reporting bias is constant over the 7-d interval. To see this, consider that the true number of infected individuals N* is related to *N* via the proportion of cases reported, *p*, such that *N* = pN*. Substituting this expression for *N* into the expression for *λ*, it is apparent that *p* cancels out. Hence, so long as *p* is approximately constant across a 7-d interval, it does not affect the estimate of growth rate.

We used a hierarchical Bayesian Gaussian regression with a log link on the weekly transmission rate. This took the form

log(λ) =α + Xβ+Zb + ε

$$\varepsilon ∼N\left(0,\sigma \right)$$

$$b∼N\left(0,\Sigma \right),$$

where λ is the growth rate of cases over one week, α is an intercept, *X* is a matrix of covariates, β is a vector of coefficients for the climate, demographic covariate *Z* is a matrix of binary dummy variables representing the political units, and *b* is a vector of coefficients describing the random intercepts for each political unit. Errors ε are normally distributed with mean zero variance σ, while political unit effects *b* had mean zero and variance Σ. Priors on α, β, ε, and Σ were weakly informative to help stabilize models as recommended in the documentation for the rstanarm R package used to fit models (39). Three chains were sampled with STAN’s no-U-turn algorithm with a burn-in of 200, and 8,000 samples thinned by a factor of 5, which was determined sufficient based on the Raftery diagnostic (40).

The full model included mean 14-d lagged temperature, mean 14-d lagged relative humidity, mean 14-d lagged absolute humidity, mean 14-d lagged UV, human population density, and proportion of the population over age 60 y. All variables were rescaled to have zero mean and unit variance to enable comparison between coefficients. We used linear terms for all variables but also considered a quadratic term for temperature, based on suggestions of modality in previous studies (10, 11, 41). Based on sensitivity analyses discussed below, we found that maximum daily UV light was a considerably better predictor than the mean (difference in Leave-One-Out Information Criterion [LOOIC] = 35), so we used the maximum in our best model. Country-level random effects were used to capture differences in policies, health care, or other locally specific behaviors. We also explored state/province-level random effects (where applicable), but country-level effects performed considerably better in all models explored, based on model selection criteria.

### Model Selection.

We were interested in developing models with high predictive ability. Thus, we performed model selection using LOOIC. This technique iteratively uses all data except for the *i*th data point to develop a model; then it predicts the left-out point, and uses the divergence between model prediction and observation to rate model performance. The sum of these divergences across all *N* data points is then converted into a standard measure of overall model performance called the LOOIC, where lower numbers indicate models that better predict left-out data (42). This model selection method has been found to excel over alternative Bayesian methods such as Deviance Information Criterion, and is especially appropriate when the objective is prediction (42).

Model selection was performed by starting with the full model and using forward and backward stepwise selection. The full model regressed the growth rate over a 1-wk window against linear terms for mean temperature, mean UV light, mean relative humidity, mean absolute humidity population density, and proportion of the population over age 60 y. We included a quadratic term for temperature based on earlier studies suggesting a decline in growth rate with temperature. We also included an interaction term between temperature and UV light to account for their correlation. All these variables were calculated in the 7-d windows preceding the interval used to calculate growth rate. During stepwise selection, we note that there were no cases of parameters trading off with one another and that coefficients for each predictor always retained the same sign and approximate magnitude regardless of which other predictors were in the model. The only exception to this was when UV light was excluded from a model that included temperature; the temperature effect dropped from positive to near zero. Hence it is important to interpret the positive effect of temperature in our best model as accounting for the effect of temperature only after UV light has been included in the models.

Once we found the best suite of predictors (excluding the quadratic temperature term, the UV light−temperature interaction, absolute humidity, and population density), we explored whether using the maximum or minimum daily values of each weather variable, and 7- versus 14-d lagged intervals, improved LOOIC. The only case where we found significant model improvement compared to the biweekly means was for maximum UV light over both 7- and 14-d intervals. Since the 14-d interval improved model performance most (based on LOOIC), we chose that as the summary statistic for UV light. Notably, for all other weather variables, there was negligible difference in LOOIC when we used weekly versus biweekly means, and hence we used biweekly values for all variables for simplicity.

### Sensitivity Analysis.

Sensitivity analysis for a variety of model decisions was conducted to determine whether our key finding—the relation between COVID-19 growth rate and temperature, UV light, and relative humidity—was affected by any of our decisions. In all cases that follow, the median of the temperature coefficient was positive, with a 95% credible interval sometimes overlapping zero and sometimes not, depending on the model. In all cases, the median and 95% credible interval for UV was negative. In all cases, the 95% intervals for relative humidity and population density always overlapped zero, but the medians were always negative and positive, respectively. The quadratic temperature term never improved the model, indicating that there was no support for a unimodal response to temperature.

Sensitivity to a number of data preparation steps was assessed. During data preparation, we considered the two, three, and four worst weeks (highest lambda) following *t*_*0*_, as well as the first 2, 3, and 4 wk following *t*_*0*_. We note that the first 3 wk and the worst 3 wk coincided for 554/592 data points used for model fitting. We chose different cutoffs (20, 40, 60) for numbers infected to account for the difficulty in determining the time when spread became local rather than imported. Due to the strong control measures in place in China by the time our dataset begins (January 22, 2020), we also compared our best model with and without data from China and found no qualitative change in outcomes.

### Coefficients over Time.

To explore how our inference about different weather factors may have changed over time as the virus approaches a geographic and environmental equilibrium (which it may still not be at), we fit a model each day since February 1, 2020, accumulating infection data up until the most recent date of analysis. This analysis can illustrate 1) how earlier studies may have inferred a negative dependence of growth on temperature, 2) the uncertainty inherent in earlier estimates of temperature dependence, 3) the disequilibrium between COVID-19 and the environment early in its spread, and 4) the smaller credible intervals, and hence increasing confidence, in our model based on more recent data. Note that the model used to illustrate this pattern 1) used the first (rather than the worst) 3 wk following *t*_*0*_ to accumulate data as early as possible and thus reflect decisions made in earlier studies, and 2) used polity (rather than country) effects because the data in early February were predominantly from China, and thus country effects could not be fit. Although early data gaps meant that we could not precisely replicate previous analyses with this exercise, we obtained similar outcomes using this model for the present analysis (again indicating model robustness). As well, this exercise demonstrated how conclusions from earlier studies may have arisen, even with our more refined model, but based on a longer time series.

### Projections.

Future predictions of the potential growth rate by month were made by projecting our highest-performing model according to LOOIC. Importantly, we reinforce that our predictions pertain to the possible growth rate in the absence of social distancing or other control measures, because it is based on a model fit with infections that occurred primarily before precautionary policies were implemented. Note that, even if a policy was implemented on, for example, March 14, we expect that infections reported in the next 2 wk were initiated before the policy began. Hence, we predict the underlying contribution of weather to future COVID-19 growth. Importantly, these predictions reflect what would happen if other control measures are relaxed and the natural dynamics of infection can begin again in a population with little resistance. Currently, governments are deciding when and how to relax control measures, often under the assumption that weather will lessen the potential for spread in the upcoming months. Thus, whereas we do not presume to predict the actual future growth rate of COVID-19, we do hope to capture the potential maximum growth rate in order to inform the relative risks of alternative control strategies.

To make future projections, we obtained monthly mean temperature and relative humidity weather data from 2015 to 2019 from the same data source as above, under the assumption that these recent years are representative of what to expect in the coming months. Notably hotter or cloudier (lower UV light) days in the coming months would suggest higher growth rates than we predict. UV data were not available in a monthly aggregation, so we obtained the 3-hourly data and aggregated it to monthly values. Human population was assumed to remain constant. We projected the models without random effects (or, equivalently, at the mean value of 0), as we were reluctant to assume that country-level policies, reporting, or health care potential will remain the same in the future. We expect that different country-level effects will dominate in the future, but predicting these offsets is beyond the scope of this study.

### Caveats.

As with any predictive study, we seek to use the best available data and understanding of mechanisms to develop possible projections that make clear underlying decisions and uncertainty. Ultimately, such predictions must be treated with appropriate caution given the limited understanding of SARS-CoV-2 virus, human resistance to it, and its transmission dynamics at this time. Thus, while we seek to inform decisions, those decisions must also recognize the inherent uncertainty in any predictive model, but especially in the context of limited information. Future data will ultimately be the arbiter of these predictions, and thus good predictive modeling will require repeated bouts of model validation, revision, and reprojection as we learn more about this virus.

In particular, we await mechanistic information on viral physiology and human resistance, to move beyond the correlative approach taken here by necessity. Mechanistic models apply insights about an organism’s intrinsic biology using parameters often collected from careful experimental manipulations. However, in the absence of this information, correlative models can predict near-term dynamics with accuracy (43, 44). Bayesian approaches like ours can integrate both mechanistic and correlative knowledge as these pieces of information become available.

One thing that we do not account for in our model is human behavior and control measures. By modeling maximum growth rate and using a threshold number of cases, we restrict our analyses to the period during which the disease expanded quickly, following the beginning of community transmission but before major control measures were implemented. For instance, most countries began implementing national control measures in mid-March, which would influence infections recorded into early April, based on a 14-d window for symptoms to emerge. Hence, we chose to limit our dataset to records before April 7. However, we note that, following early April, growth rates are expected to be much lower due to control measures, and these will continue to be important to reduce growth rates below the potential values we predict here which do not account for control.

We used available insights about SARS-CoV-2, related viruses, and observations of COVID-19 dynamics to select a list of factors that likely influence it. Although we purposefully limited these variables to reflect our best knowledge and to avoid overfitting, certainly, other climate and epidemiological factors are likely missing from the model. Future studies should consider embedding these climate insights into epidemiological models that include human demography, immunity, movement, behaviors, medical capacity, and control efforts (4).

## Supplementary Material

Supplementary File

## Data Availability

Data and code used in this analysis are publicly available in Github at https://github.com/cmerow/MerowAndUrban_COVID19_PNAS_2020.

## Change History

October 15, 2020: The license for this article has been updated.
