# Supplementary material for: Seasonality and uncertainty in global COVID-19 growth rates
Source: Proc Natl Acad Sci U S A. 2020 Oct 13;117(44):27456–64. doi: 10.1073/pnas.2008590117 (PMC7959558; doi:10.1073/pnas.2008590117)
Supplement: Supplementary File [file pnas.2008590117.sapp.pdf]

www.pnas.org/cgi/doi/10.1073/pnas.2008590117

Supplementary Information for

Seasonality and uncertainty in global COVID-19 growth rates

Cory Merow & Mark C. Urban

Corresponding author: Cory Merow
Email: cory.merow@gmail.com

This PDF file includes:

Figures S1 to S7
Tables S1

Table S1: Alternative models and parameter estimates. A comparison of models considered is shown with median parameter values and followed by 95% credible intervals. Models are ordered by LOOIC (leave-one-out information criterion). Of the 153,664 models that could be constructed from the collection of variables we considered (each weather variable summarized by the minimum, mean, and maximum over a 14- or 7-day window) we considered those which were pertinent to our specific a priori hypotheses about the roles of temperature, humidity, and UV on growth of COVID-19 infections. Variable names are abbreviated as: Temp=temperature, UV=ultraviolet light, RH=relative humidity, AH=absolute humidity, PopDensity=Population density, PropOver60=proportion of population over

	looic	Intercept	TempLag14mean	TempLag14mean_sqrd	tempUV	uvLag14mean	uvLag14max	uvLag14min	rhLag14mean	rhLag14max	rhLag14min	ahLag14mean	popDensity	propOld
1	-1361.95	-2 (-2.09,-1.92)	0.23 (0.15,0.32)				-0.44 (-0.53,-0.36)		-0.05 (-0.11,0)					-0.07 (-0.14,0)
2	-1360.65	-1.92 (-2.04,-1.81)	0.07 (-0.11,0.26)	-0.1 (-0.2,0)	0.03 (-0.07,0.14)		-0.45 (-0.54,-0.35)		-0.13 (-0.23,-0.03)			0.2 (-0.01,0.39)	0 (-0.1,0.08)	-0.09 (-0.16,-0.01)
3	-1360.26	-1.99 (-2.08,-1.91)	0.23 (0.15,0.31)				-0.44 (-0.52,-0.35)				-0.05 (-0.1,0)			-0.08 (-0.15,-0.01)
4	-1357.66	-2 (-2.09,-1.92)	0.22 (0.14,0.3)				-0.42 (-0.51,-0.34)			-0.03 (-0.09,0.04)				-0.07 (-0.14,-0.01)
5	-1355.65	-1.96 (-2.07,-1.86)	0.23 (0.1,0.37)	-0.06 (-0.15,0.03)	0.02 (-0.08,0.12)		-0.41 (-0.5,-0.32)					-0.02 (-0.13,0.1)		-0.1 (-0.17,-0.02)
6	-1355.21	-1.94 (-2.05,-1.84)	0.15 (-0.02,0.31)	-0.08 (-0.17,0.01)	0.03 (-0.07,0.14)		-0.43 (-0.52,-0.34)				-0.07 (-0.13,0.01)	0.09 (-0.08,0.25)	0 (-0.09,0.08)	-0.1 (-0.17,-0.02)
7	-1354.06	-1.93 (-2.04,-1.82)	0.15 (-0.01,0.33)	-0.09 (-0.18,0.01)	0.02 (-0.08,0.12)		-0.42 (-0.5,-0.33)			-0.07 (-0.16,0.02)		0.06 (-0.1,0.22)	0.01 (-0.08,0.08)	-0.09 (-0.17,-0.02)
8	-1326.78	-1.9 (-2.01,-1.79)	-0.04 (-0.22,0.13)	-0.12 (-0.23,-0.02)	0.12 (0.02,0.22)	-0.4 (-0.51,-0.3)			-0.22 (-0.32,-0.11)			0.22 (0.02,0.42)		-0.08 (-0.16,0)
9	-1325.48	-1.9 (-2,-1.79)	-0.04 (-0.21,0.15)	-0.12 (-0.22,-0.01)	0.12 (0.02,0.23)	-0.4 (-0.51,-0.3)			-0.21 (-0.33,-0.11)			0.22 (0.02,0.42)	0.01 (-0.07,0.09)	-0.08 (-0.15,0)
10	-1324.58	-1.9 (-1.98,-1.82)				-0.42 (-0.52,-0.32)			-0.2 (-0.29,-0.12)			0.18 (0.08,0.27)		-0.07 (-0.15,0)
11	-1323.18	-1.9 (-1.98,-1.83)	0.04 (-0.12,0.19)			-0.42 (-0.52,-0.32)			-0.19 (-0.29,-0.08)			0.14 (-0.04,0.31)		-0.07 (-0.15,0)
12	-1323.14	-1.93 (-2.04,-1.83)	0.05 (-0.1,0.2)		0.02 (-0.04,0.09)	-0.42 (-0.52,-0.32)			-0.18 (-0.29,-0.07)			0.12 (-0.05,0.3)		-0.07 (-0.14,0.01)
13	-1322.68	-1.91 (-2.02,-1.81)	-0.01 (-0.18,0.18)	-0.11 (-0.21,-0.01)	0.12 (0.02,0.23)	-0.37 (-0.47,-0.27)			-0.21 (-0.32,-0.1)			0.2 (-0.01,0.39)		
14	-1321.60	-1.95 (-2.05,-1.85)	0.14 (0.06,0.22)		0.03 (-0.03,0.1)	-0.4 (-0.49,-0.3)			-0.12 (-0.19,-0.06)					-0.07 (-0.14,0.01)
15	-1321.20	-1.87 (-1.97,-1.77)	0.01 (-0.16,0.18)	-0.03 (-0.09,0.03)		-0.41 (-0.52,-0.31)			-0.2 (-0.31,-0.09)			0.18 (-0.01,0.38)		-0.08 (-0.16,-0.01)
16	-1321.19	-1.93 (-2.04,-1.84)	0.13 (0.05,0.22)	-0.07 (-0.17,0.02)	0.1 (0,0.2)	-0.37 (-0.47,-0.27)			-0.12 (-0.19,-0.05)					-0.07 (-0.15,0.01)
17	-1319.78	-1.91 (-1.99,-1.84)	0.14 (0.07,0.22)			-0.39 (-0.48,-0.29)			-0.12 (-0.19,-0.05)					-0.07 (-0.15,0)
18	-1319.36	-1.94 (-2.05,-1.84)	0.07 (-0.08,0.23)		0.03 (-0.03,0.1)	-0.39 (-0.49,-0.3)			-0.18 (-0.29,-0.07)			0.11 (-0.07,0.28)		
19	-1318.85	-1.96 (-2.06,-1.86)	0.16 (0.08,0.23)		0.04 (-0.02,0.11)	-0.37 (-0.46,-0.28)			-0.13 (-0.19,-0.06)					
20	-1318.03	-1.9 (-1.98,-1.83)	0.06 (-0.09,0.21)			-0.39 (-0.48,-0.29)			-0.19 (-0.3,-0.09)			0.14 (-0.04,0.3)		
21	-1317.92	-1.91 (-2,-1.82)	0.14 (0.06,0.22)	-0.01 (-0.06,0.05)		-0.39 (-0.48,-0.28)			-0.12 (-0.19,-0.05)					-0.07 (-0.16,0)
22	-1308.67	-1.95 (-2.06,-1.85)	0.17 (0.03,0.31)	-0.07 (-0.17,0.02)	0.1 (0,0.19)	-0.3 (-0.38,-0.21)						-0.08 (-0.21,0.03)		-0.07 (-0.14,0)
23	-1269.25	-1.93 (-2.03,-1.83)	0.03 (-0.11,0.16)	-0.15 (-0.25,-0.05)	0.19 (0.09,0.29)			-0.15 (-0.23,-0.07)				-0.03 (-0.15,0.09)		-0.02 (-0.09,0.05)
24	-1268.38	-1.91 (-2.03,-1.81)	-0.05 (-0.23,0.14)	-0.17 (-0.29,-0.07)	0.2 (0.1,0.31)			-0.17 (-0.26,-0.08)	-0.07 (-0.18,0.04)			0.06 (-0.14,0.26)	0 (-0.09,0.08)	-0.02 (-0.1,0.05)
25	-1256.54	-1.9 (-1.97,-1.83)	0.03 (-0.05,0.1)					-0.18 (-0.25,-0.1)	-0.03 (-0.1,0.03)					-0.02 (-0.09,0.05)
26	-1254.52	-1.9 (-2,-1.79)	-0.05 (-0.23,0.15)	-0.19 (-0.3,-0.09)	0.18 (0.09,0.28)				0.02 (-0.08,0.12)			-0.01 (-0.22,0.19)		0.01 (-0.06,0.08)

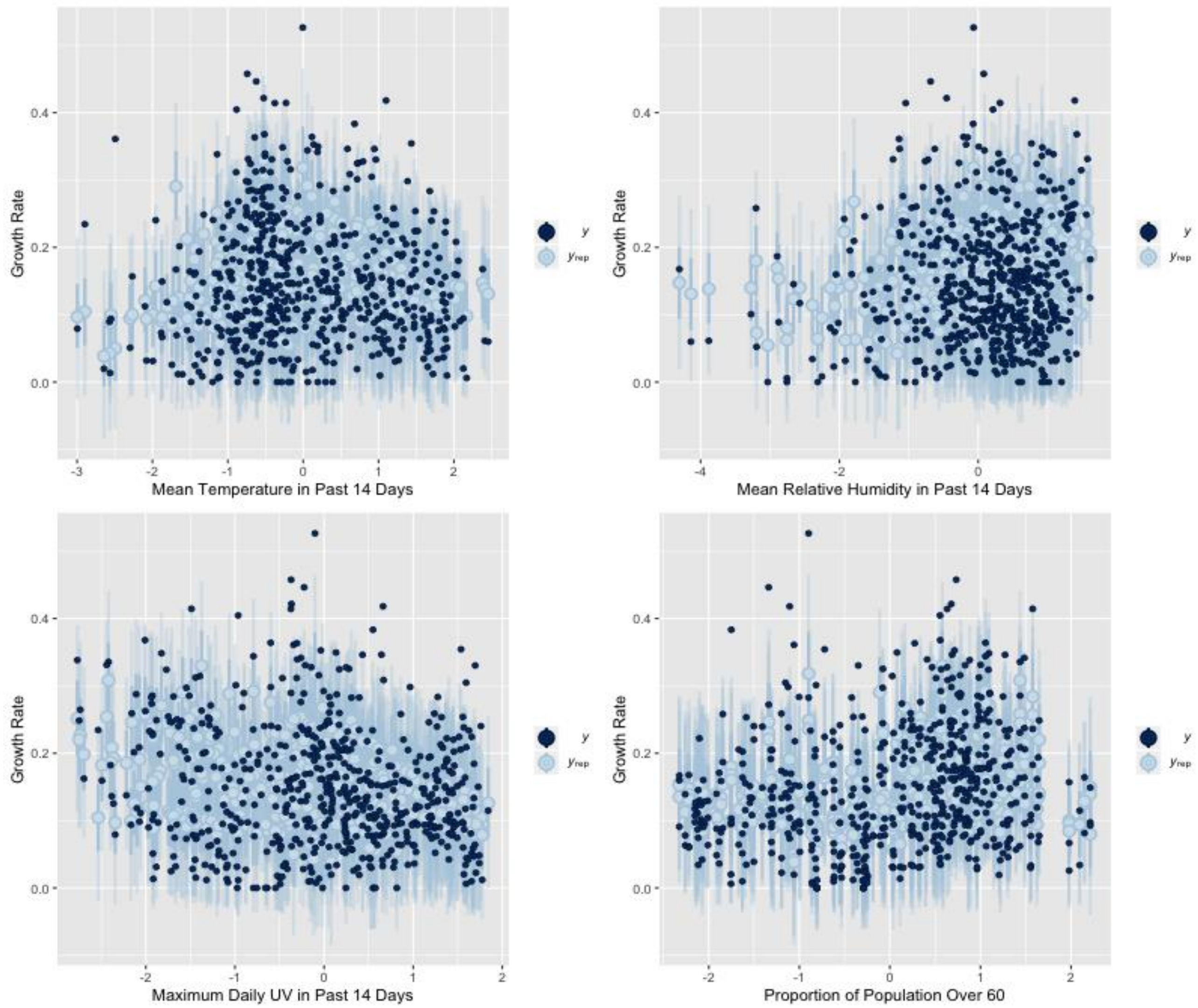

Fig. S1. Posterior predicted probabilities of growth rate reflect weak trends with environment and high uncertainty in predictions.

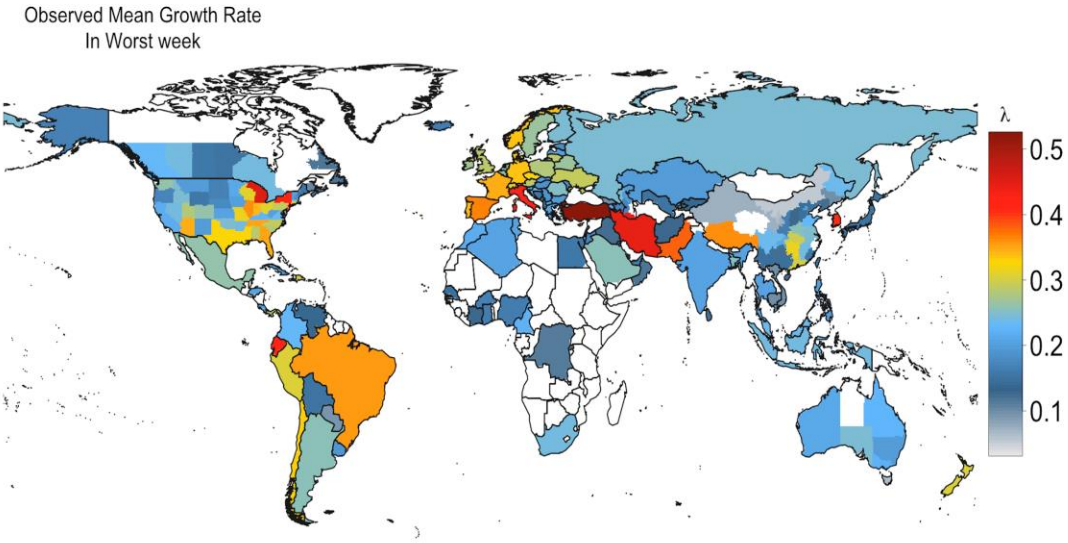

Fig. S2. A map of the growth rate of COVID-19 cases during the worst week considered in this study (Jan 22, 2020 - April 13, 2020).

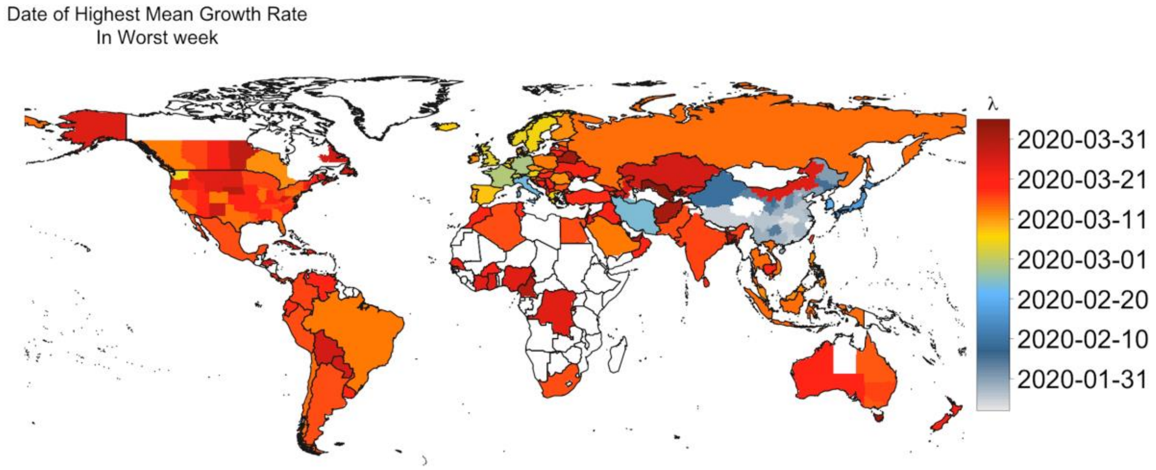

Fig. S3. A map of the date (first day of the week) of the mean growth rate of COVID-19 cases during the worst week (as shown in Fig. S2) considered in this study (Jan 22, 2020 - April 13, 2020).

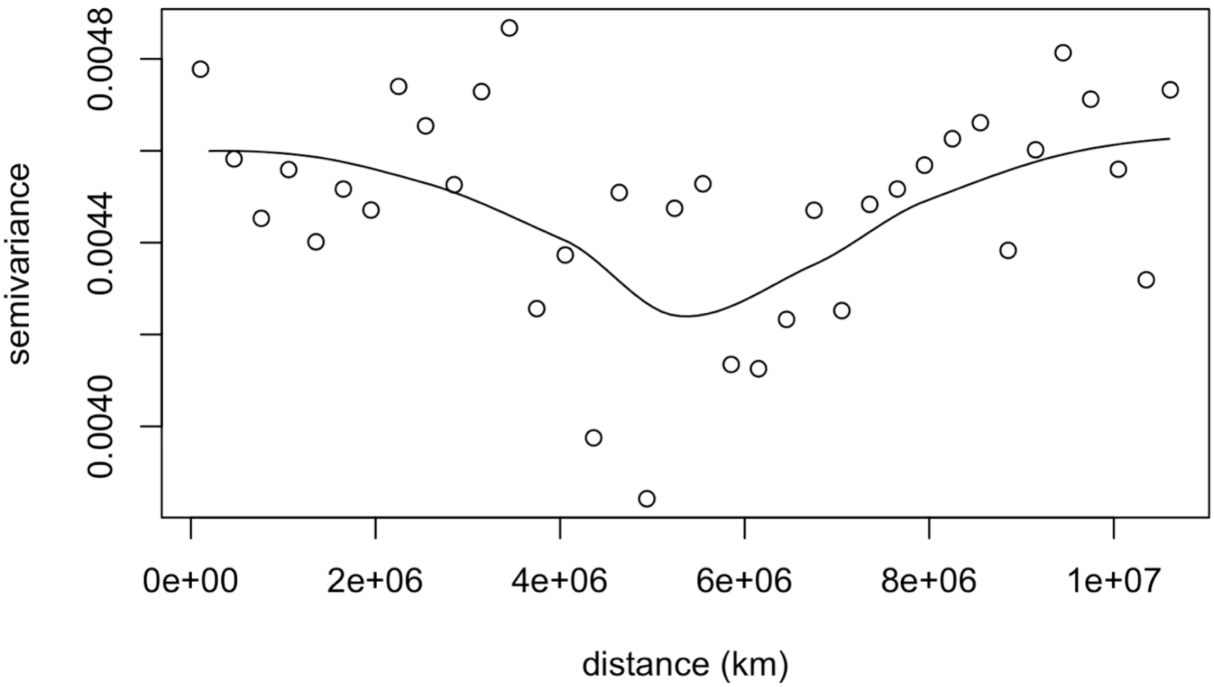

Fig. S4. Semivariogram of the model residuals from the best model, used to confirm that there is no spatial dependence apparent.

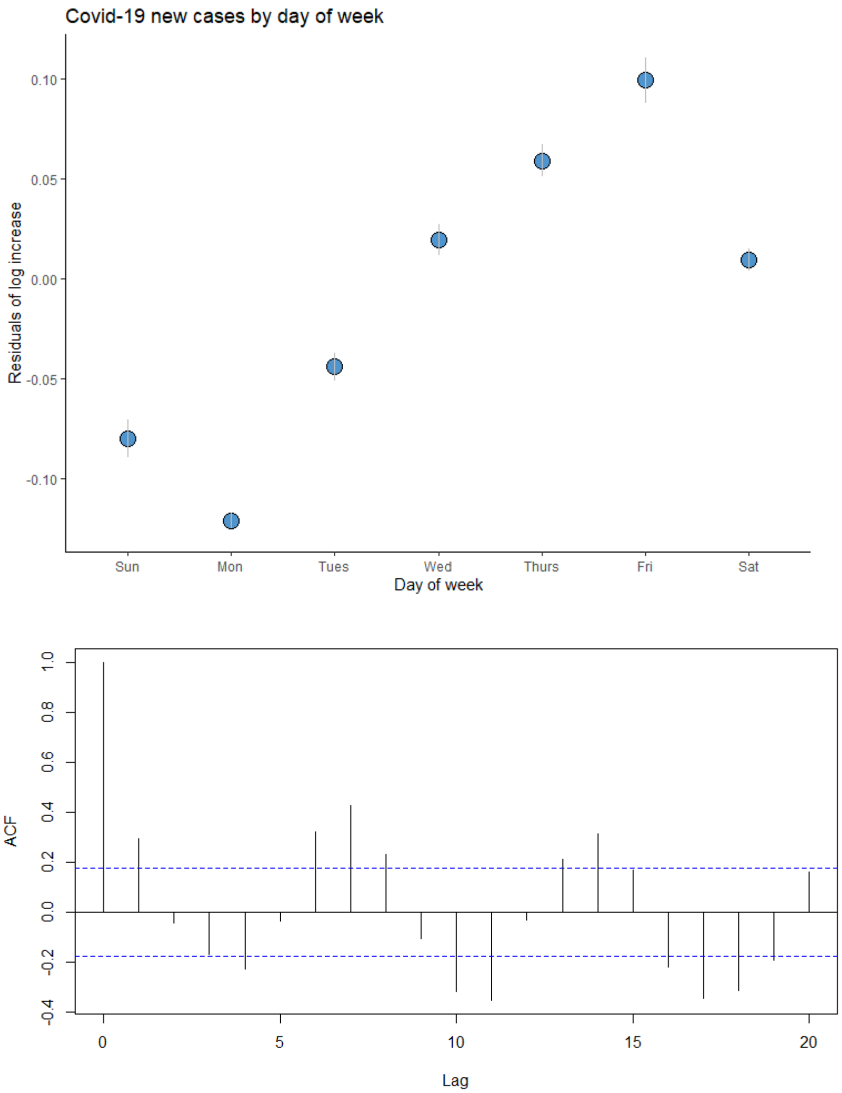

Figure S5. Top, Mean and standard error of the mean of the detrended residuals of log COVID-19 daily increase in new cases by day of week. Detrending was done to remove the temporal trend of increasing number of cases during the study period and was performed by calculating the residuals of a cubic spline generalized additive model of daily log COVID-19 increase by day. The Bayesian 95% credible intervals for Saturday through Wednesday did not overlap with Friday. **Bottom,** Plot of temporal autocorrelation (ACF) of same data by lag time in days reveals a strong weekly pattern as indicated by the significant peaks at t = 7 and t = 14 days. Lines that exceed the dotted line are significantly different from zero. We restricted the analysis to the period 3/12/20 – 7/14/20 because preliminary analyses suggested high day-to-day variance due to early vagaries in reporting.

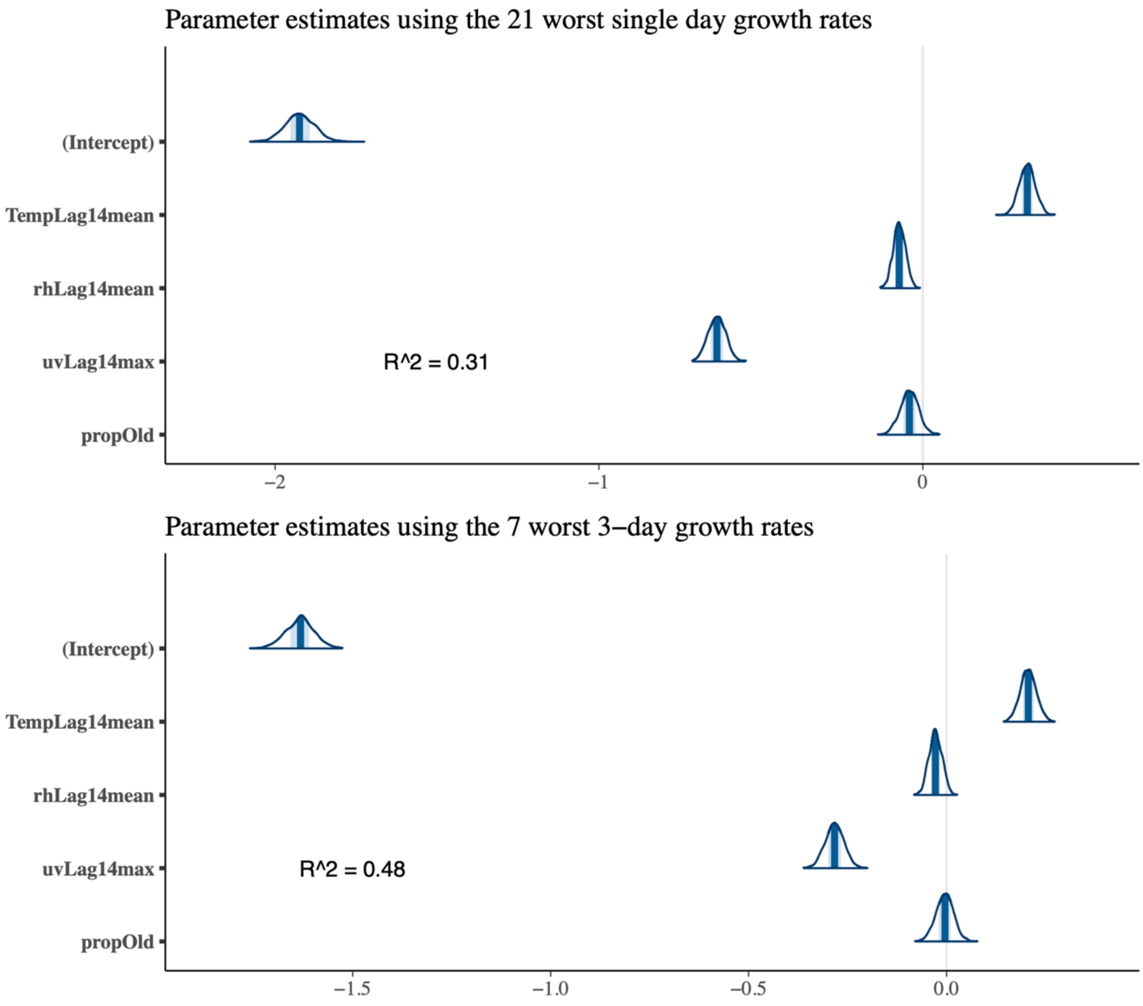

Figure S6. In the main text, we calculated the growth rate of COVID-19 infections over 1-week intervals in each polity separately, under the assumption that testing rates were, on average, roughly similar over the interval within a given polity. To relax this assumption and examine whether our results are robust to it, we built two additional variations on our best model (Fig. 2a, main text). In the first variation, we modeled the growth rates of the worst 21 1-day intervals and in the second variation we modeled the worst 7 3-day intervals. These models reflect relaxed assumptions of shorter time intervals over which testing rates were assumed to remain similar. Importantly, the coefficient values, and their relationships to one another are essentially unchanged compared to the weekly models in the main text, hence our predictions -which derive from these coefficient estimates - are robust to this variation. Not surprisingly, the median R^2 was lower using 1-day intervals, reflecting additional noise likely driven by regional reporting patterns on particular days of the week (e.g., reporting is lowest on Mondays and highest on Fridays, globally).

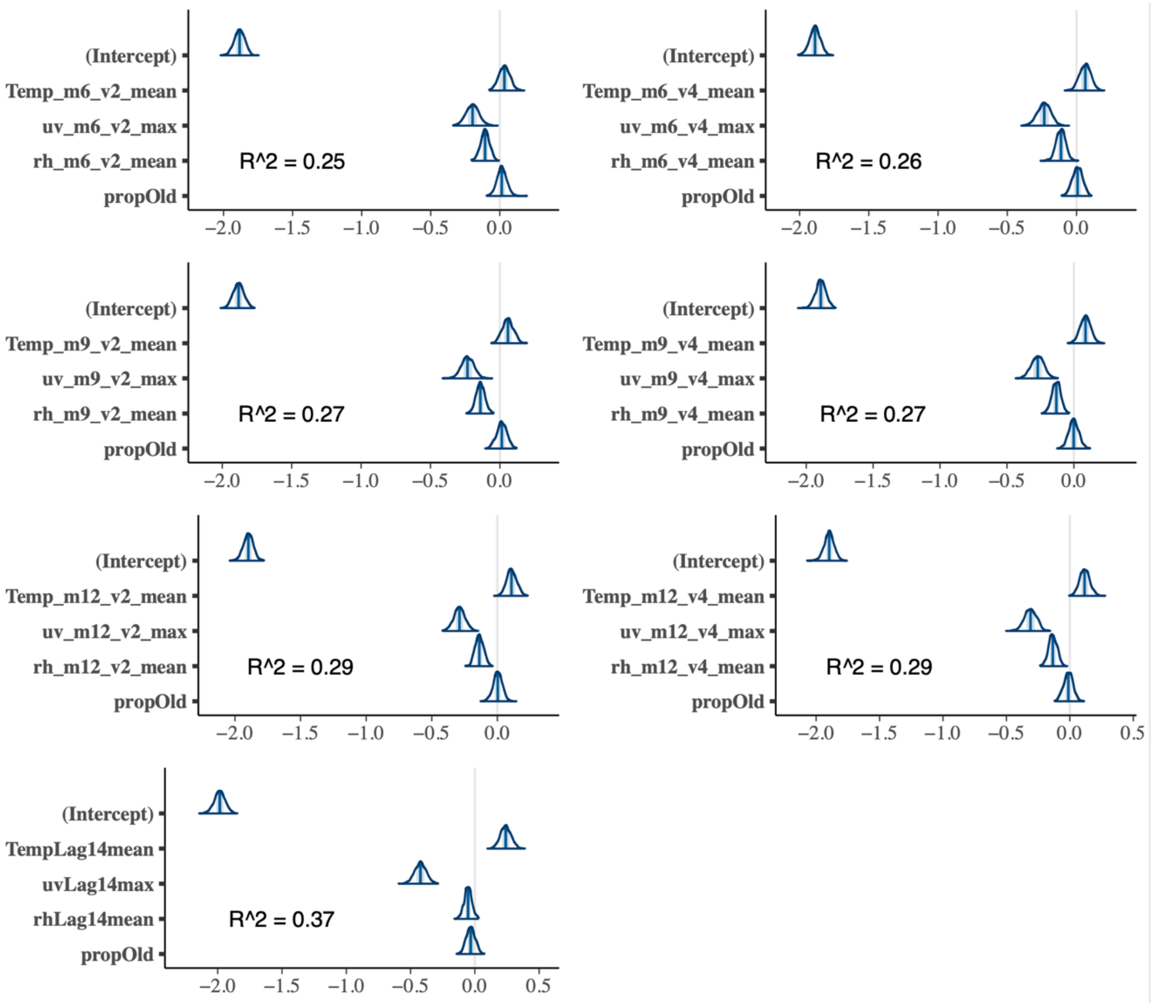

Figure S7. Coefficient estimates from various ways of calculating weather covariates. Notably all show the same pattern as the model in the main text (Fig. 2a, and bottom left panel here) due to the strong temporal autocorrelation in weather. In the main text, we present weather covariates that were calculated as the (unweighted) average of the 14-day interval preceding a given COVID-19 growth rate estimate. This reflects the assumption that each day is equally important in determining growth rate. For example, if weather influences if an individual was infected on a given day, then each day is equally likely. One might also hypothesize that the weather is most important on a particular suite of days rather than across the entire interval, e.g., because the typical time for symptoms to emerge is 4-5 days, there may be a few days lag before an individual has access to testing, and results of tests can take multiple days to be reported. To examine sensitivity in different assumptions about the most important time interval for weather and different times when an individual might be infected, we built six additional variations on our best model (Fig. 2a). We calculated the same three weather covariates used in the best model (mean daily temperature, mean daily relative humidity, and maximum daily UV) over a 21-day lagged interval as Gaussian weighted averages centered on 6, 9, and 12 days (denoted m6, m9, m12 in

variable names in the figure), each with standard deviation of 2 or 4 (denoted v2 and v4 in variable names in the figure). All models using the Gaussian weighted variables showed the same basic patterns, because typically high temporal autocorrelation in weather means that all variants of weighting schemes will result in similar covariate values (see Fig. S8). It is interesting that higher explanatory power was found ($R^2=0.37$) when using the unweighted averages, perhaps indicating that some cumulative effect of weather, rather than instantaneous values on the day of infection, is important.

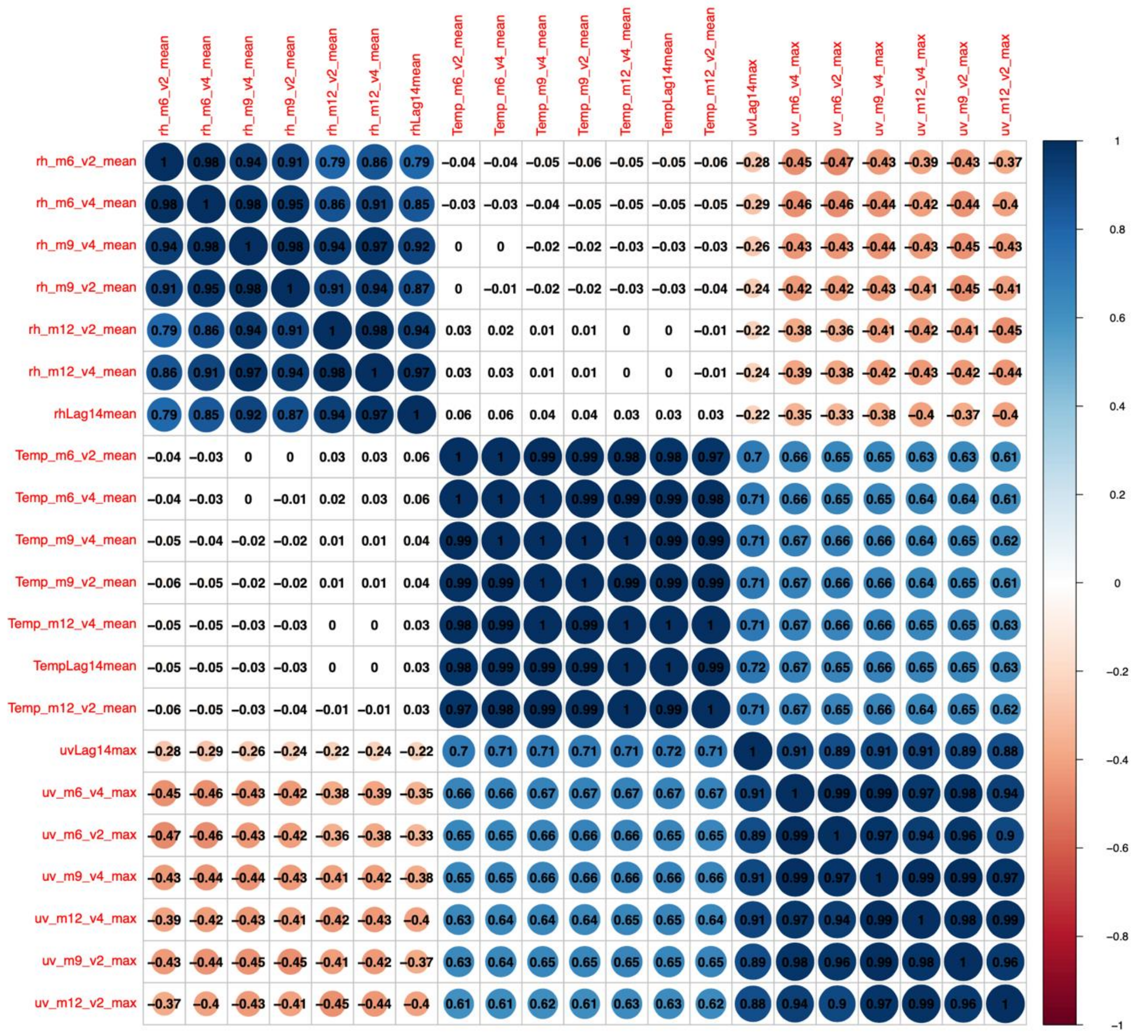

Figure S8. High correlations are apparent among different ways of calculating lagged weather covariates. Gaussian weighted variables showed the same basic patterns as the uniformly weiged 14-day lagged variables, because typically high temporal autocorrelation in weather at each location means that all variants of weighting schemes will result in similar covariate values. Variable names are coded as : Temp=mean daily temperature, rh=mean daily relative humidity, uv= maximum daily UV. Codes m6, m9, and m12 correspond to Gaussian weighting centered on days 6, 9, or 12 days before a recorded positive test. Codes v2 and v4 reflect the standard deviations of 2 or 4, respectively, for these weights. It is evident from these very high correlations within each variable block (7 x 7 blocks on the diagonal) that high temporal autocorrelation in weather means that variable assumptions about the timing of reporting and infection during a 14-day interval does not exhibit sufficient variation to affect our results appreciably. Rather, the important weather variation is among polities, not among daily differences within polities.
